# Effects of the Developmental Regulator BOLITA on the Plant Metabolome

**DOI:** 10.3390/genes12070995

**Published:** 2021-06-29

**Authors:** Hugo Gerardo Lazcano-Ramírez, Roberto Gamboa-Becerra, Irving J. García-López, Ricardo A. Chávez Montes, David Díaz-Ramírez, Octavio Martínez de la Vega, José Juan Ordaz-Ortíz, Stefan de Folter, Axel Tiessen-Favier, Robert Winkler, Nayelli Marsch-Martínez

**Affiliations:** 1 Cell Identity Laboratory, Biotechnology and Biochemistry Department, CINVESTAV-IPN Irapuato Unit, Irapuato 36824, Mexico; Hugo.Lazcano@cinvestav.mx (H.G.L.-R.); david.diaz@cinvestav.mx (D.D.-R.); 2 Laboratory of Biochemical and Instrumental Analysis, Biotechnology and Biochemistry Department, CINVESTAV-IPN Irapuato Unit, Irapuato 36824, Mexico; roberto.gamboa@inecol.mx; 3 Red de Biodiversidad y Sistemática, Instituto de Ecología A.C. Carretera Antigua a Coatepec 351, El Haya, Xalapa, Veracruz 91073, Mexico; 4 Genetic Engineering Department, CINVESTAV-IPN Irapuato Unit, Irapuato 36824, Mexico; irving.garcia@cinvestav.mx (I.J.G.-L.); axel.tiessen@cinvestav.mx (A.T.-F.); 5 Advanced Genomics Unit (UGA-Langebio), CINVESTAV-IPN, Irapuato 36824, Mexico; ra.chavez.montes@gmail.com (R.A.C.M.); octavio.martinez@cinvestav.mx (O.M.d.l.V.); jose.ordaz.ortiz@cinvestav.mx (J.J.O.-O.); stefan.defolter@cinvestav.mx (S.d.F.); 6 Institute of Genomics for Crop Abiotic Stress Tolerance, Texas Tech University, Lubbock, TX 79409, USA

**Keywords:** transcription factor, metabolic fingerprinting, developmental regulation, phenylpropanoid pathway, glucosinolates, global expression analysis

## Abstract

Transcription factors are important regulators of gene expression. They can orchestrate the activation or repression of hundreds or thousands of genes and control diverse processes in a coordinated way. This work explores the effect of a master regulator of plant development, BOLITA (BOL), in plant metabolism, with a special focus on specialized metabolism. For this, we used an *Arabidopsis thaliana* line in which the transcription factor activity can be induced. Fingerprinting metabolomic analyses of whole plantlets were performed at different times after induction. After 96 h, all induced replicas clustered as a single group, in contrast with all controls which did not cluster. Metabolomic analyses of shoot and root tissues enabled the putative identification of differentially accumulated metabolites in each tissue. Finally, the analysis of global gene expression in induced vs. non-induced root samples, together with enrichment analyses, allowed the identification of enriched metabolic pathways among the differentially expressed genes and accumulated metabolites after the induction. We concluded that the induction of BOL activity can modify the Arabidopsis metabolome. Future work should investigate whether its action is direct or indirect, and the implications of the metabolic changes for development regulation and bioprospection.

## 1. Introduction

Although the genome sequence of most model organisms has been completed, the understanding of how this genetic code is executed during development to build a complete organism with a specific phenotype is still a long way off [1]. It is not possible to fully predict the function and participation of a protein in all biological processes only by knowing and studying the basis of its sequence [2].

The study, at any level of biological organization, of living beings that presents information that is qualitative or quantitative must be considered a phenotypic study (for example, sizes, shapes, colors, concentrations), because it constitutes the result of the materialization of the genetic program in the conditions of observation and measurement [3]. The molecules that are produced by an organism represent the metabolic phenotype, though changes in the concentrations or presence or absence of most molecules cannot be detected by visual inspection. Plants produce a large number of molecules with different basic life functions, including maintenance, growth, development, and response to the environment that surrounds them [4,5]. These low molecular weight molecules often have a major impact on the yield and quality of crops.

Generally, these metabolites are classified as primary and secondary. Secondary metabolites are also referred to as specialized metabolites or natural products [5]. Primary metabolites are essential for the growth and development of a plant. Specialized metabolites are considered non-essential but important for survival under special conditions by maintaining a balance with the environment. Furthermore, primary metabolites are highly conserved; that is, they can be found throughout the plant kingdom compared to the “secondary,” some of which may be specific to certain taxonomic groups [6].

The comprehensive study of metabolic profiles of organisms is called ‘Metabolomics.’ Gas or liquid chromatography coupled to mass spectrometry is currently the gold standard for analyzing complex mixtures from plant tissues [7,8]. Combining different techniques and ionization methods allows a broad coverage for the detection, quantification, and identification of compounds. Targeted strategies permit the sensitive and selective monitoring of low-abundance metabolites such as hormones [9]. In contrast, un-targeted methods serve to explore metabolic changes and phenotypes in biological experiments [10]. Still, the unequivocal identification of metabolites from mass spectrometry data is not trivial. Usually, an informatics workflow with several data processing and statistical analysis tools is required to analyze mass spectrometry-based metabolomics data [11].

In addition, there are different types of protein in an organism capable of influencing or modulating the metabolic phenotype of an organism, such as certain enzymes and transcription factors.

Transcription factors are regulatory proteins that modulate the expression of groups of genes through specific DNA sequence binding domains and protein–protein interactions. They interact with the transcriptional machinery, chromatin remodeling proteins, and/or other transcription factors and can act as activators or repressors of gene expression by modulating the rate of synthesis of messenger RNA from their target genes [12]. Therefore, they play an important role in determining the phenotypes that an organism can present in response to endogenous development programs and different internal or external conditions or stimuli, at different levels. While enzymes act directly by altering the metabolic flow, transcription factors can simultaneously affect the expression of multiple genes encoding enzymes, transporters, downstream transcription factors, or other genes that respond to metabolites.

The transcription factor BOLITA (BOL) [13], also called DORNROESCHEN-LIKE (DRNL) [14] and ENHANCER OF SHOOT REGENERATION 2 (ESR2) [15], belongs to the superfamily of transcription factors of APETALA2/Ethylene Responsive Factor (AP2/ERF) transcription [16]. The members of this superfamily contain one or two AP2/ERF domains [16,17,18].

Its expression is limited to young tissues, starting from the regions in meristems that will give rise to a new organ (initially a primordium), especially leaves and floral organs [19]. Once the structure of the new organ is established, its levels begin to decline. Its overexpression in *Arabidopsis thaliana* causes abnormal plant development. The defects of these plants are visible to the naked eye because their size is severely affected. Their organs are reduced in size caused by a decrease in the size and number of their cells.

Additionally, it promotes the reprogramming of cell identity by inducing the formation of green calli (shoot-like) in the main and adventitious roots of *A. thaliana* and ectopic floral structures in tobacco [13]. The effect of its overexpression on shoot formation and plant regeneration has also been reported in in vitro cultures obtained from root explants [15]. However, this effect has been reported for a very small number of genes.

Advances have been made in elucidating the role that this transcription factor has in the development of Arabidopsis. However, little is known about the effect it may have on plant metabolism. This knowledge can both support the bioprospection of this transcription factor and be useful in understanding the mechanisms, some probably unexpected, by which it modulates plant development. Therefore, in this work, we investigated whether the induction of the developmental regulator BOL could change the metabolic fingerprint of Arabidopsis through metabolomic and transcriptomic analyses and their correlation.

## 2. Materials and Methods

### 2.1. Plant Material and Growth Conditions

Two *A. thaliana* genotypes were used: wild-type Col-0 and an inducible line of the BOL transcription factor, 35Spro:ESR2-ER (also named as ESR2-ER), which was kindly shared by Dr. Eva Sundberg [15,20]. The seeds were surface sterilized using a chlorine gas sanitization protocol. Seeds of the two genotypes were germinated at 22 °C under long-day conditions (16 h light/8 h dark) on agar plates (0.8% *w*/*v*) of 1× Murashige and Skoog medium [21] supplemented with vitamins, and 1% (weight/volume) sucrose.

### 2.2. Tissue Sampling for Direct Liquid-Injection Electrospray Ionization Mass Spectrometry (DLI-ESI MS)

To perform the DLI-ESI MS [22] analysis, whole seedlings were transferred to 24-well ELISA plates seven days after germination. Five seedlings were placed in each well that contained 2 mL of MS medium prepared according to the following treatments: inducer medium with ten µM of β-estradiol, medium with the solvent (ethanol) but without the inducer (“mock” or control), and “blank” medium without supplements. The tissue for the metabolomic analysis was collected in quintuplicate at four different times after the transfer (24, 48, 72, 96 h).

### 2.3. Tissue Sampling for Ultra-High Performance Liquid Chromatography Coupled to Electrospray Ionization Mass Spectrometry (UPLC-ESI MS) and Transcriptomic Analysis

An in vitro plant-growth protocol based on the method published by Hétu et al. (2005) [23] was modified to obtain enough root biomass for the UPLC-ESI MS analysis. *A. thaliana* seeds were germinated on agar plates on an anti-aphid mesh (0.75 mm × 0.75 mm opening). Seven days after germination, the meshes were transferred to 125 mL flasks containing 10 mL of liquid MS medium, supplemented with 2% sucrose. After ten days, the volume of the flask was increased to 15 mL, and the total volume was replaced by fresh medium 17 and 24 days after the transfer of the mesh. 24 days after germination, the medium was replaced by fresh medium without sucrose and supplemented with β-estradiol or ethanol. Aerial and root tissue were collected 96 h after induction. Aerial tissue samples were only used for metabolomic analyses, and root samples were used for metabolomic and transcriptomic analyses.

### 2.4. Tissue Preprocessing for Metabolite Extraction

Tissue preparation (seedlings, shoots or roots) for the analysis was performed as follows: three washes were carried out with sterile deionized water, which was then frozen with liquid nitrogen. Next, the frozen tissue was dried by lyophilization (in a Virtis Sentry 2.0 lyophilizer; SP Industries Inc., Warminster, PA, USA) at −50 °C and 100 mTorr for one week. Next, the dry tissue was thoroughly macerated in a pearl mill (Retsch brand MM 400 Mixer Mill) until a fine powder was obtained. Finally, 15 mg of dry tissue was taken for the extraction of metabolites for DLI-ESI MS and UPLC-ESI MS.

### 2.5. Metabolite Extraction for Undirected Metabolomic Studies

The dry tissue powder was resuspended in 1 mL in a methanol: water: formic acid mixture (75.00%: 28.85%: 0.15%) [24]. Subsequently, the samples were sonicated for 20 min and centrifuged at 10,000 rpm for 5 min. The supernatant was collected and filtered using 0.22 µm membranes (Titan3™ Nylon Syringe Filters-Thermo Fisher Scientific, Waltham, MA, USA).

### 2.6. DLI-ESI MS

For the DLI-ESI MS analysis, the filtered extract was diluted 1: 100 in the solvent mixture methanol: water: formic acid (75.00%: 28.85%: 0.15%). The samples were analyzed in positive mode, on a ZQ 2000 quadrupole analyzer (Waters Corporation, Milford, MA, USA). Ten μL were injected using the internal pump of the equipment. The capillary voltage was adjusted to 3 kV, the cone voltage at 30 kV, and the extractor voltage was 3 kV. Continuous spectra were collected in a mass-to-charge (*m*/*z*) range of 15 to 2000, with a duration of 2 min per run, with scan times of 1 s.

### 2.7. UPLC-ESI MS

UPLC-ESI MS analysis was performed on a UPLC-Acquity chromatograph (Waters) coupled to an SYNAPT G1 mass spectrometer (Waters). Aliquots of 10 μL of each sample were analyzed on an ACQUITY UPLC CSH C18 column (1.7 μm; 2.1 × 50 mm; Waters). The mobile phase consisted of a mixture of solvent A (water with 0.1% *v*/*v* formic acid) and solvent B (acetonitrile with 0.1% *v*/*v* formic acid). The column temperature was 30 °C. The flow was adjusted continuously at 3 µL/min.

The acquisition of ESI MS was performed in positive mode, and the conditions were as follows: capillary voltage, 2.2 kV; cone voltage, 30 V, and exhaust voltage, 4 V.

### 2.8. Metabolomic Data Analysis

For the analysis of data acquired by DLI-ESI MS, the files in raw format (*.raw) were converted to universal format (*.mzML) using the ProteoWizard package (Kessner et al., 2008) and processed in the statistical language R [25]. They were used together with the libraries “MALDIquant,” “MALDIquantForeign,” “vegetarian,” “pheatmap,” “colorspace” and “pvclust” for the comparison of metabolic fingerprints with the construction of dendrograms and heatmaps. The R script is available as Supplementary Material (Data S1). The data obtained from the UPLC-ESI MS system were preprocessed using the local R library “XCMS” [26]. The consensus alignment matrix was extracted in comma-separated values format (*.csv) for statistical analysis using the MetaboAnalyst tool [27]. Samples were median-normalized and log transformed. Through t-test analysis differentially enriched features were detected. Correction for multiple testing was performed in MetaboAnalystR (SSPA R package) by setting the FDR (False Discovery Rate)-adjusted *p*-value threshold to 0.05. Only the quality ions present in all samples were used for analysis. The putative identification of the metabolites was achieved by using the SPIDERMASS tool [28], using the *A. thaliana* database previously reported by Sotelo-Silveira et al. (2015) [29], using the following parameters: mass error: 0.01 *m*/*z*; ionization mode (adducts): [M+H]^+^, [M+K]^+^, [M+Na]^+^; SpiderMass DB search: on; ChemSpider.com search: off; de-novo formula builder: off; isotope distribution fit (IDF): off.

### 2.9. RNA Extraction for RNA-seq

RNA isolation was performed from root tissue of samples obtained in liquid culture 96 h after induction. Three biological replicates were obtained for each treatment. The tissue was frozen in liquid nitrogen and stored at −80 °C. Frozen samples were macerated in microcentrifuge tubes. Total RNA extraction was carried out with the Quick-RNA MiniPrep kit (Zymo Research, Irvine, CA, USA), following the manufacturer’s specifications. Subsequently, RNA purity and concentration were evaluated in a NanoDrop 2000 equipment (Thermo), and its integrity was visualized by agarose gel electrophoresis.

### 2.10. RNA-seq

Global messenger RNA sequencing was performed by Macrogen’s “RNA-seq” service in South Korea. In summary, Illumina TruSeq RNA libraries were prepared, and 100 bp (Paired-End) sequences were obtained from each of them on a HiSeq 4000 platform. Between 12 and 14 million reads per library were obtained. The data are deposited at the NCBI, BioProject ID: PRJNA739193.

### 2.11. Transcriptomic Data Processing

Raw fastq files were processed with the Trimmomatic tool [30] version 0.36 in paired mode with the parameters ILLUMINACLIP:TruSeq2-PE.fa:2:30:10 LEADING:3 TRAILING:3 SLIDINGWINDOW:4:20 MINLEN:50. The resulting fastq files were quality-checked using FastQC. Transcript abundance quantification was done using kallisto (v0.43.1; [31]) versus the Araport11 [32] cdna blastset available at The Arabidopsis Information Resource. Differential expression was performed with gene-level summarization using tximport [33] as described in the vignette available at Bioconductor [34,35], and edgeR [36] using a paired sample design, an induced vs. non-induced contrast, and a likelihood ratio test with default parameters. We considered genes with an absolute logFC value of 1.5 or higher and an FDR-adjusted *p*-value of 0.05 or lower as differentially expressed.

## 3. Results

### 3.1. BOL Activity Affects the Metabolic Fingerprint of A. thaliana Seedlings

First, we investigated the effects of the increased activity of the BOL transcription factor at the metabolomic level using whole seedling tissue to know whether it could influence the metabolic fingerprint of *A. thaliana.* For this, a comparison was made between wild-type Col-0 seedlings and an inducible line where the entrance to the nucleus, and therefore the transcriptional regulation activity of this transcription factor, was induced by β-estradiol, called ESR2-ER (35Spro:ESR2-ER).

The treatments to which the two genotypes were subjected were: (1) induction conditions (the medium was supplemented with β-estradiol at a final concentration of 10 μM); (2) “mock” treatment (or control, medium supplemented with the same volume of ethanol, but without β-estradiol); and (3) medium without additional treatment (“blank” medium). This experimental design was applied to compare metabolic fingerprints and identify global changes specifically caused by the action of this transcription factor and not merely a reaction caused by the inducing agent or the solvent used. For this first analysis, complete 7-day-old seedlings were used. Five samples were taken for each treatment and genotype.

After an undirected extraction of metabolites as described in Materials and Methods, the samples were analyzed by DLI-ESI MS [37] to obtain a global panorama of the variations in the metabolic signature after the induction of BOL activity. DLI-ESI MS is a high-throughput method for classifying samples according to their chemical profile. In contrast to UPLC-ESI MS, the compounds are not chromatographically pre-separated, and therefore the identification and quantification capabilities of this method are limited. However, mass fingerprinting with DLI-ESI MS enables the direct and fast classification of metabolic phenotypes and the statistical evaluation of genetic or environmental effects on plant metabolism [37].

The terminology used for each treatment is shown in Table 1. The analysis showed no significant differences in the metabolic fingerprint caused by the transcription factor during the first three sampling times after induction (24, 48, and 72 h).

**Figure 1:**
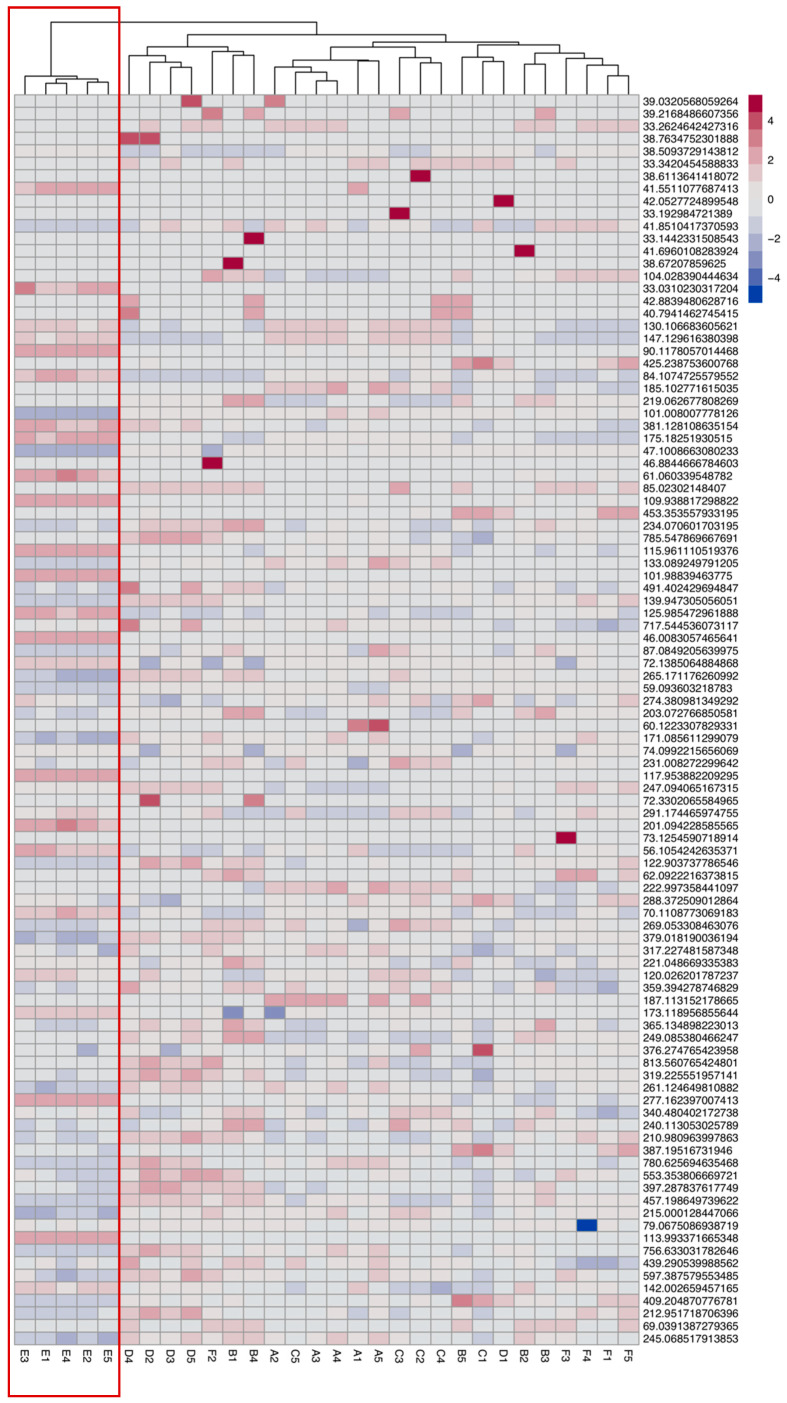
Heat map of the metabolic fingerprint generated with the 100 most abundant DLI-ESI MS ions of the samples analyzed 96 h after the induction of the transcription factor. At the top, a hierarchical grouping dendrogram made using Euclidean agglomeration and distances established by Ward’s method. The red box indicates the grouping of samples with probability >95%. The letters correspond to the following samples: A: p35S:ESR2-ER+No treatment; B: Col-0+No treatment; C: p35S:ESR2-ER+Ethanol; D: Col-0+Ethanol; E: p35S:ESR2-ER+ Ethanol+β-ESTRADIOL; F: Col-0+Ethanol+β-ESTRADIOL. The numbers correspond to the sample number (replicas). Color intensity represents relative accumulation. The greater the red hue, the greater the relative accumulation; the greater the blue hue, the less relative accumulation.

Interestingly, for the samples obtained 96 h after the treatment, there was an evident effect. The dendrogram generated by clustering the abundance data of the 100 most abundant ions showed a clear grouping of the samples corresponding to the inducible genotype treated with the inducing agent, separating all the biological replicas in a group independently from the rest of the samples (Figure 1). Therefore, there is a clear effect of the induction of the transcription factor in the metabolomic footprint, detected 96 h after induction with the methods used.

### 3.2. Differentially Accumulated Metabolites in Root and Aerial Tissues

**Figure 2:**
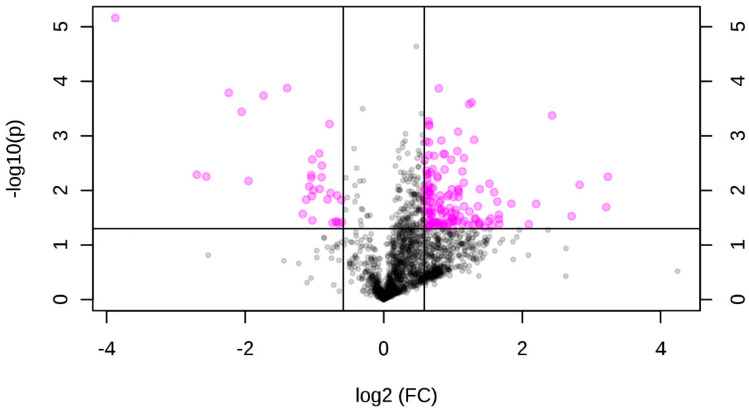
Volcano plot showing the differential accumulation of ions in induced vs. non-induced root tissue samples. The *X*-axis shows the base 2 logarithms of the proportion of change in accumulation or “fold change,” and the *Y*-axis shows the negative value of the log_10_ of the “*p*” value; the lines parallel to the *Y*-axis represent a value of “fold change” = 1.5. The line parallel to the *X*-axis represents a value of “*p*” = 0.05. The pink color shows the 165 differentially accumulated ions (*p* ≤ 0.05 and “fold change” ≥ 1.5) in root tissue. The black color indicates the ions that did not show differential accumulation.

**Figure 3:**
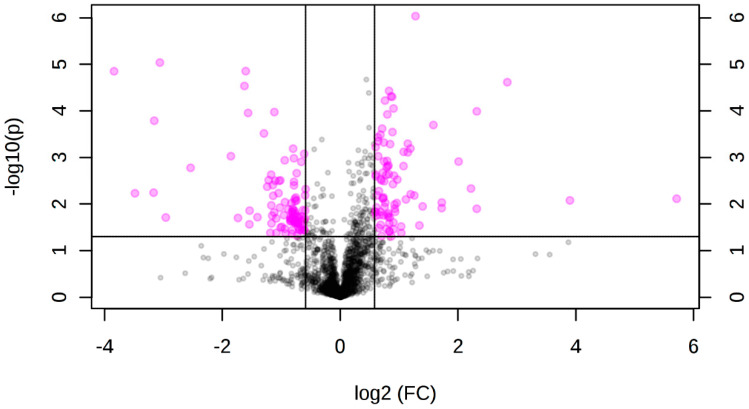
Volcano plot showing the differential accumulation of ions in induced vs. non-induced aerial tissue samples. The *X*-axis shows the base 2 logarithms of the “fold change,” and the *Y*-axis shows the negative value of the base 10 logarithms of “*p*.” The lines parallel to the *Y*-axis represent a log_2_ “fold change” = 1.5. The line parallel to the *X*-axis represents a value of “*p*” = 0.05. The pink color shows the 184 ions that differentially accumulated (*p* ≤ 0.05 and “fold change” ≥ 1.5) in aerial tissue. The black color indicates the ions that did not show differential accumulation.

After observing that the metabolite fingerprint of the seedlings was changed 96 h after the induction of the transcription factor, we sought to explore the differential accumulation of metabolites in different tissues. Therefore, we used seedlings 96 h after induction and dissected them to obtain aerial tissues and roots separately. After processing and extraction, these samples were analyzed by UPLC-ESI MS. After data processing using the R version of the xcms tool, considering all the data sets as a whole, a total of 1771 ions considered of quality were obtained and quantified in all samples. When comparing induced to non-induced root samples, a total of 165 ions presented significant accumulation differences (*p* ≤ 0.05 and proportion of change in their accumulation or “fold change” ≥1.5) in the induced tissue, of which 134 presented an increase, and 31 a decrease, in their accumulation (Figure 2). In aerial tissue, a total of 184 ions showed significant differential accumulation (*p* ≤ 0.05 and “fold change” ≥1.5) upon induction, of which 98 showed an increase and 87 a decrease in their accumulation (Figure 3).

### 3.3. Identification of Differentially Accumulated Metabolites

For the putative identification of differentially accumulated metabolites, the “SPIDERMASS” tool [28] was used, using as a reference a database of previously reported *A. thaliana* metabolites [29]. From these differentially accumulated metabolites, the putative identification of 11 metabolites in the aerial part and 29 in root tissue could be carried out (Table 2 and Table 3). Interestingly, six of these were found in both aerial and root tissues.

### 3.4. RNA-seq Analysis

**Figure 4:**
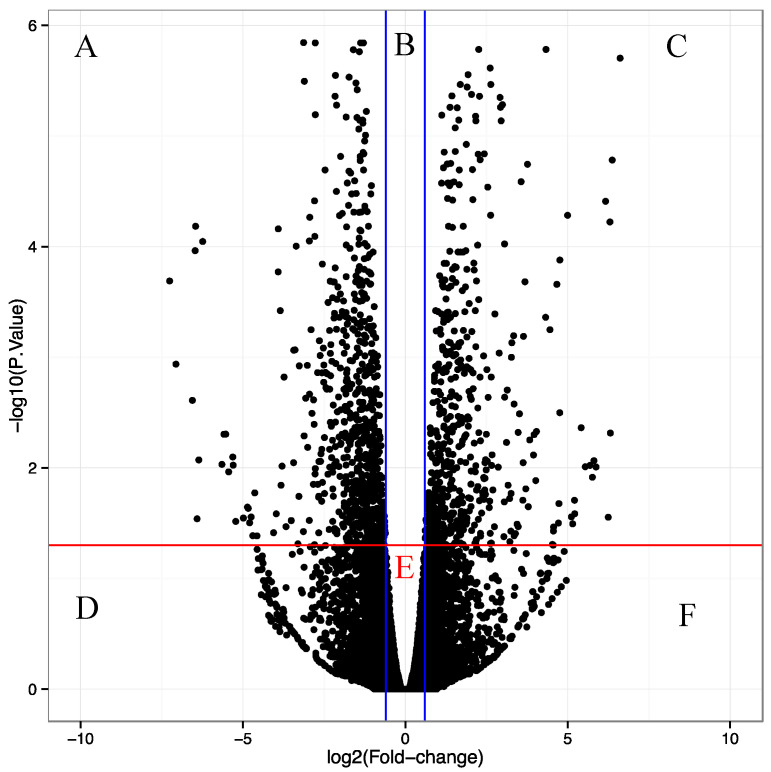
Volcano plot showing 14,708 genes obtained in the transcriptomes, mapped within the *A. thaliana* genome. The base 2 logarithms of the fold change are shown on the *X*-axis. The negative value of the base 10 logarithms of the “*p*” value is shown on the *Y*-axis. Thus, the blue lines parallel to the *Y*-axis represent a “fold change” value = 1.5, and the red line parallel to the *X*-axis represents a “*p*” value = 0.05. The A and C sections are the only ones that include the differentially expressed genes (*p* ≤ 0.05 and “fold change” ≥ 1.5).

The nature of the detected metabolites in the untargeted metabolomic analyses depends on the extraction method employed. No extraction method can obtain all metabolites in a sample, and several are lost during extraction. Therefore, we sought to identify the changes in the transcript accumulation of metabolism-related genes to obtain a broad perspective of the possible metabolic changes triggered by the transcription factor. To obtain a global view of the changes in gene expression at the time at which the differential accumulation was detected, we performed transcriptomic analyses in the same samples of induced vs. non-induced roots 96 h after induction. Total RNA was isolated from three biological replicas of root tissue from the same samples used for UPLC-ESI MS metabolite analysis. Reads were processed, and *A. thaliana* transcript expression was quantified and summarized at the gene level. 14,708 genes had evidence of expression, of which 1413 were differentially expressed (FDR-adjusted *p*-value ≤ 0.005 and logarithm base 2 of the “fold change” ≥1.5). Of these, 800 were downregulated and 613 upregulated in roots where transcription factor activity was induced (Figure 4).

### 3.5. Enrichment Analyses

We sought to integrate the metabolomic and transcriptomic data to further explore the candidate metabolites and metabolic pathways modulated by the induction of BOL. For this, we used the pathway enrichment tool from the KEGG database (https://www.genome.jp/kegg/, accessed on 30 March 2020 [38]). In the case of the aerial part tissue, for which only data from a few metabolites was available, no significant enrichment (*p* ≤ 0.05) was found for any of the pathways present in the database. However, the analyses of differentially accumulated metabolites and differentially expressed genes found in induced roots revealed enrichment in different metabolic pathways, presented in Table 4 and Table 5.

**Figure 5:**
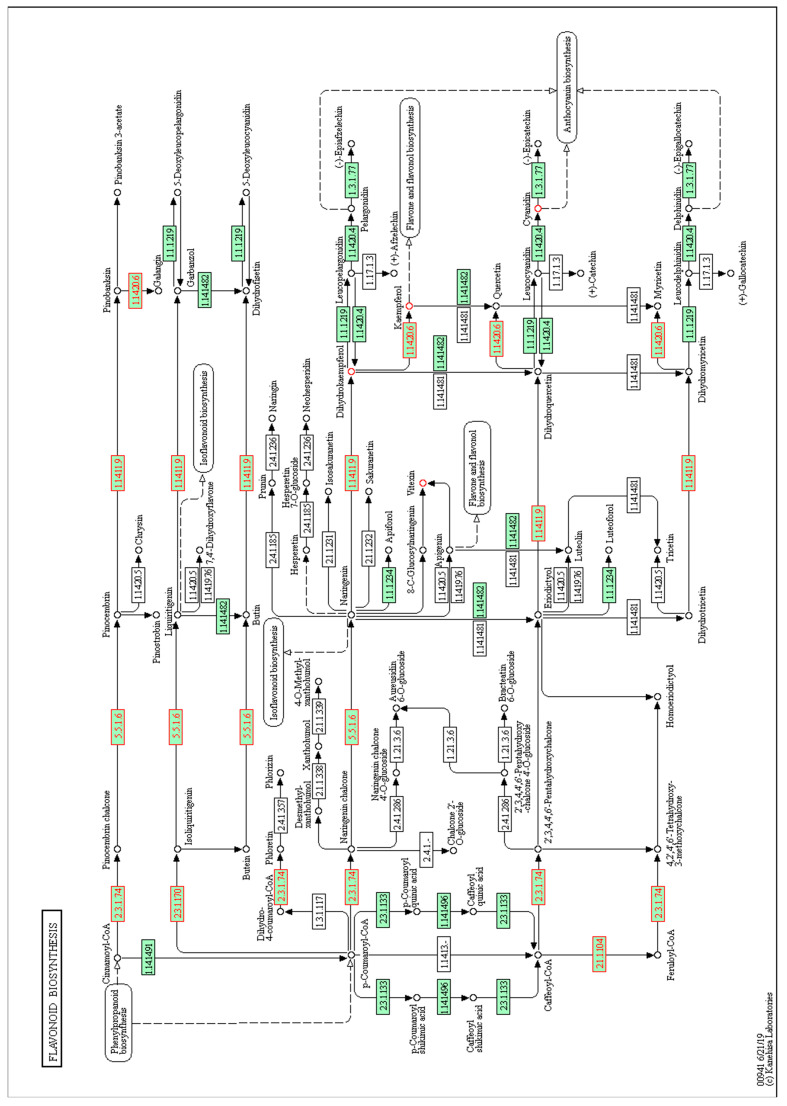
Map of the KEGG phenylpropanoid biosynthetic pathway depicting the differentially expressed genes and differentially accumulated metabolites after BOL induction. Arrows represent enzymatic steps. Genes belonging to the reference pathway are shown in green squares, and the differentially expressed genes mapped within the pathway are marked with red numbers and borders. The circles represent metabolites, and red circles mark the differentially accumulated metabolites.

Interestingly, some common pathways showed enrichment in both the root metabolomic and transcriptomic data, such as the phenylpropanoid biosynthesis route (including large compound groups such as flavonoids and lignins, among others). Table 6 includes the phenylpropanoid-related differentially expressed genes and differentially accumulated metabolites upon BOL induction. Their localization in the pathway is depicted in Figure 5. All the differentially accumulated metabolites of this pathway showed increased accumulation upon BOL induction. However, the case of the genes was different because while one of the genes was upregulated, the others were downregulated, which could suggest a feedback mechanism that affects the genes in the pathway.

**Figure 6:**
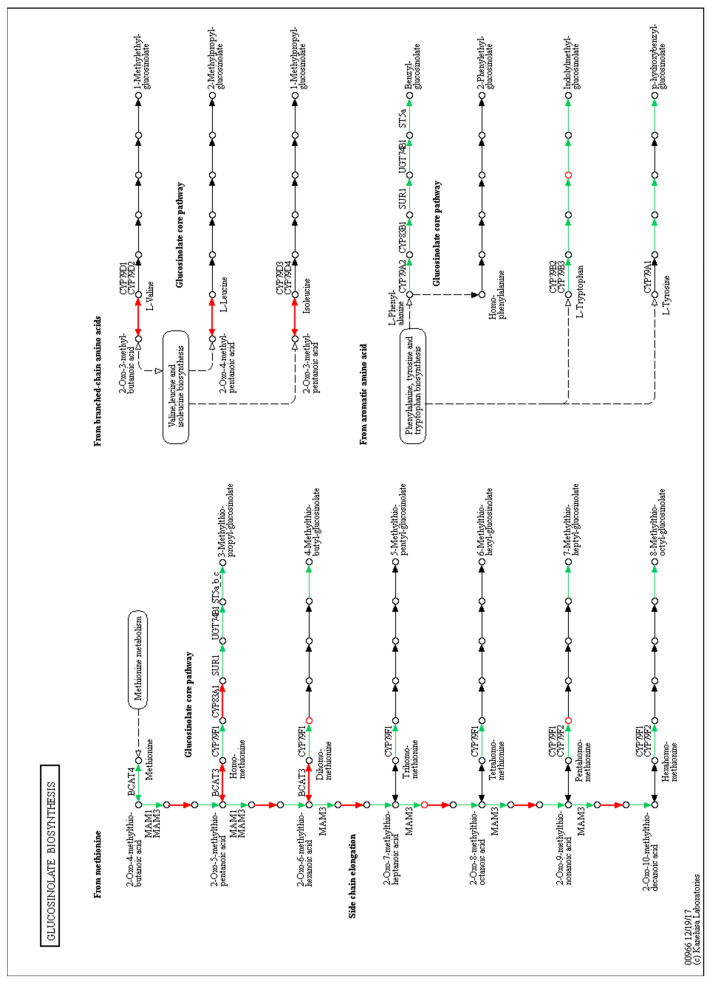
Map of the KEGG glucosinolate biosynthesis pathway depicting the differentially expressed genes and differentially accumulated metabolites after BOL induction. Arrows represent enzymatic reactions, and the names of the corresponding enzymes are indicated above them. Red arrows indicate enzymes coded by differentially expressed genes upon BOL induction. The circles represent metabolites, and red circles mark the differentially accumulated metabolites.

There were other interesting pathways enriched using the metabolites and the transcripts dataset in the KEGG database. As an example of one of these pathways, we looked for the metabolites and genes related to glucosinolate metabolism, a Brassicaceae-specific pathway. The differentially accumulated metabolites and transcripts related to this pathway are listed in Table 7 and mapped in the pathway in Figure 6. In this case, some differentially accumulated metabolites showed increased accumulation, while others decreased. The same was observed for transcript accumulation.

After observing the changes in the accumulation of different metabolites and transcripts, we concluded that the induction of the BOL transcription factor has an evident effect on the metabolome of Arabidopsis seedlings. Interestingly, the differentially accumulated metabolites and transcripts data presented an enrichment of some specific pathways. It will be interesting, in further work, to investigate whether this is due to the direct regulation of the transcription factor or an indirect regulation or response of the plant towards homeostasis, and what is their relevance in the developmental processes that BOL controls.

## 4. Discussion

The transcription factor BOL can reprogram plant cell identity, producing conspicuous changes in the morphology and physical characteristics of different tissues [11,13]. In this work, we investigated the impact of this developmental regulator at the metabolic level. We found that evident changes in the metabolomic footprint can be detected 96 h after BOL induction. At this time after induction, the morphological and physical changes in the tissues are not yet so evident, suggesting that the metabolic changes may start to occur together and probably not as a consequence of the morphological and physical phenotype. It is likely that these changes start early after the induction but are very subtle and therefore are not detected until after 96 h. The induction mechanism in the system used in this work relies on the entrance of the transcription factor to the nucleus. After entering the nucleus, the transcription factor can then activate or repress genes, and the effects of these changes in genetic regulation will, therefore, first impact transcription. Then, processes such as RNA processing and trafficking, degradation, and translation must occur to produce a sufficient amount of proteins, in this case, enzymes or metabolic regulators, which may need also to be post-translationally activated. Finally, the metabolites should reach a certain accumulation level in order to be detected. This could explain the time required to detect differences. The time after which the changes are observed does not allow us to discern whether these changes are due to direct or indirect regulation of the transcription factor of the different pathways or adjustments made in different cells to respond to the developmental program triggered by the transcription factor. Transcriptome analyses performed at earlier times and direct binding assays such as chromatin immunoprecipitation (ChIP) or Yeast 1-Hybrid (Y1H) could help discern this better.

Interestingly, a different number of metabolites changed in the aerial or root tissues upon BOL induction. This could be due to differences in the epigenetic and accessibility status of the regulatory regions of genes in the two tissues or to the presence or absence of other factors in each tissue, among other possibilities. Nevertheless, some metabolites presented changes in their accumulation upon BOL induction in both tissues. Therefore, it will be interesting to further investigate the role of these metabolites in the phenotype, natural function, and general reprogramming of tissues exerted by the transcription factor.

To further strengthen the metabolomic data, we also analyzed the changes in gene expression in roots after the induction of the transcription factor. We chose roots because this tissue presents the most evident changes when the transcription factor is induced, and a higher number of metabolites presented differential accumulation. We focused on those associated with metabolic pathways using the KEGG enrichment analysis tool to explore whether an enrichment in specific metabolic pathways could be detected from the genes that were found to be differentially expressed. We also used this tool to investigate whether the differentially accumulated metabolite data obtained from the root and aerial tissues also enriched specific metabolic pathways. No enrichment was detected in the differentially accumulated metabolites from aerial tissues, probably because their number was very low. However, we cannot rule out that different extraction and analysis methods would lead to finding enrichment in certain pathways. In contrast, the differential accumulation data obtained from both the metabolomic and the transcriptomic data of root tissues were enriched for specific metabolic pathways. Among them, we focused on the metabolites and genes that were differentially present in the induced roots that belonged to two pathways, flavonoids and glucosinolates.

### 4.1. Effect of BOL on the Phenylpropanoid Biosynthesis Pathway

Flavonoids are phenylpropanoids that are highly representative secondary metabolites of plants. In *A. thaliana*, slightly more than 50 different related molecules have been identified [39]. They have been reported to play roles in nodulation, fertility, defense, and protection against UV radiation [40]. Furthermore, these compounds also participate in the modulation of plant development as endogenous regulators of auxin transport [41]. A total of five differentially expressed genes and five differentially accumulated metabolites were found within the flavonoid biosynthesis pathway (Table 6 and Figure 5). Of the members of this family of compounds, the most studied flavonols are kaempferol and quercetin and, among anthocyanins, cyanidin [40]. In this study, cyanidin and kaempferol were putatively identified as differentially accumulated metabolites, increasing their relative concentration in the roots of plants with higher activity of the transcription factor BOL.

Kaempferol and its derivatives have been considered flavonoids of great interest in recent years due to the variety of properties described for them, such as antioxidant capacity and anticancer, antidiabetic cardioprotective, neuroprotective, and antimicrobial activities [42]. Furthermore, in *A. thaliana,* the role of this and its derivatives in UV radiation protection and resistance to herbivory by insects has been described [43,44]. Therefore, it is very interesting to find that the accumulation of this or similar metabolites may be increased upon BOL induction. Moreover, it was recently reported that flavonoids stabilize PIN transporters, acting similarly to the commonly used auxin transport inhibitor NPA [45]. Therefore, it will be very informative to explore whether the accumulation of flavonoids plays a role in the callus forming phenotype produced by the induction of the transcription factor or its natural function in organ formation.

The phenylpropanoid pathway includes a bifurcation that leads to the synthesis of lignins or flavonoids. In the case of the differentially expressed genes, a gene coding for CAFFEOYL-COA 3-O-METHYLTRANSFERASE (CCoAMT), an enzyme involved in lignin biosynthesis, is upregulated. This gene has been reported to be expressed in different tissues [46]. A related member has also been associated with the accumulation of synapoyl malate, which protects against UV radiation [47]. Unexpectedly, genes encoding enzymes directly related to the accumulation of flavonoids were found to be downregulated (for example, TT6, TT4, and FLS1) [48,49]. This could suggest negative feedback loops between the accumulation of metabolites and the expression of genes upon BOL induction.

Overall, the data suggests that the induction of BOL caused changes in the phenylpropanoid pathway. This opens the door to performing more detailed studies about their roles in organ development and the mechanisms of action of the BOL transcription factor (for example, in modulating auxin transport), and its bioprospection potential.

### 4.2. Effect on Glucosinolate Biosynthesis

Glucosinolates are a class of secondary metabolites that contain nitrogen and sulfur in their structure and are characteristic of the Brassicaceae family of plants. They are divided into three large groups depending on the amino acid from which they are synthesized: (i) aliphatic glucosinolates (from leucine, valine, methionine, isoleucine); (ii) indole (from tryptophan), and (iii) aromatic (from phenylalanine) [50]. These compounds are usually hydrolyzed when plants are subjected to some type of biotic or abiotic stress, participating in the defense against, or adaptation, to them [51,52].

These metabolites have generated interest because they have different antioxidant [53], insecticidal [54], antidiabetic [55], and anticancer activities [56]. Interestingly, glucosinolates have been recently reported to play roles in plant development, from root development [57,58] to biomass accumulation [59] and reproductive development [60].

Changes in both glucosinolate accumulation and glucosinolate-related gene expression were observed. Among the genes that showed differential expression, there are two that code for nitrilase enzymes, *NITRILASE2* and *4* (*NIT2, NIT4*), which have been reported as participants in the degradation of indole glucosinolates [61,62]. In addition, a high overexpression of the transcript corresponding to the *BRANCHED-CHAIN AMINO ACID TRANSFERASE2 (BCAT-2)* gene was also found. This gene encodes an enzyme responsible for the degradation of branched-chain amino acids (for example, valine, leucine, and isoleucine) [63]. These amino acids are precursors to the synthesis of aliphatic glucosinolates. In addition, a close relative of this gene (e.g., *BCAT-3*) is involved in the catalysis of the final steps in the short-chain glucosinolate elongation process [64].

The enzyme ISOPROPYLMALATE ISOMERASE 2 (IPMI2) has been associated with the elongation of the methionine chain for the production of glucosinolates [65]. Therefore, it was interesting to find the expression of this gene repressed at the transcript level since the metabolomic analysis recorded a differential accumulation of two glucosinolates (e.g., 5-methylthiopentanaldoxime and 8-methylthiooctanaldoxime) whose synthesis involves the catalytic activity of this enzyme. However, it should be noted that there are still many gaps in the phenotypic information that prevent clarification of the mechanisms of synthesis and complete regulation of the biosynthesis, degradation, or accumulation of these compounds *A. thaliana* [65].

The enzyme GLUCOSINOLATE HYDROXYLASE (GSL-OH) is a dioxygenase required to synthesize 2-hydroxybut-3-enyl-glucosinolate, reported as a direct resistance factor to insects [66]. The differential expression analysis shows an increase in the expression of some of these genes. In contrast, others decrease their expression, which could be due to direct or indirect regulation or negative feedback. In summary, the data suggests that the induction of BOL affected this pathway. Interestingly, glucosinolates have recently been found to affect reproductive development [60], and BOL regulates the development of reproductive organs [14,19,67]. However, more research will be necessary to determine whether there is a connection between glucosinolates and the BOL function in reproductive development.

The changes in both the phenylpropanoid and glucosinolate pathways after the induction of the transcription factor affect different steps in these pathways. Therefore, it will be very interesting to carefully study these and other pathways identified in this study.

It is not possible to distinguish whether these changes are due to direct regulation by the transcription factor or are the result of indirect regulation. However, some of the metabolites that change could have an active role in the developmental changes observed upon transcription factor induction, acting as still unknown signals. They could also result from a response of the plant to restore homeostasis after the transcription factor has triggered other initial changes. Nevertheless, the effect in the metabolome is clear, and the data generated will certainly lead to the exploration of metabolites that were differentially accumulated and metabolic pathways that appeared to be enriched. Moreover, this kind of study can be very useful in investigating the role of metabolites in development and the potential use of developmental regulators to modulate metabolic pathways.

## Figures and Tables

**Table 1:** Labels used in the comparative metabolic fingerprint experiment.

Label	Genotype	Treatment
A	p35S:ESR2-ER	No treatment
B	Col-0	No treatment
C	p35S:ESR2-ER	Ethanol
D	Col-0	Ethanol
E	p35S:ESR2-ER	Ethanol + β-ESTRADIOL
F	Col-0	Ethanol + β-ESTRADIOL

**Table 2:** Putatively identified metabolites with differential accumulation in root tissue after induction of the transcription factor. The table includes the information of the *m*/*z* for each molecule, the theoretical form of ionization considered, the name in the ChemSpider database, the ID within this database, the corresponding ID in the KEGG database (if present in the database), the “fold change” (the ratio between the root induced (RI) and root control tissue (RC)), and the *p*-value.

*m/z*	Ionization Mode	Name in ChemSpider	ID ChemSpider	ID KEGG	“Fold Change” (RI/RC)	*p*-Value
116.0723	[M+H]+	L-Proline	128566	C00148	1.59	0.0240
124.0420	[M+H]+	Isonicotinic acid	5709	C00253	1.71	0.0058
145.0323	[M+Na]+	Pyridine-2-aldoxime methochloride	10302871		2.96	0.0447
147.0587	[M+H]+	ketopantoic acid	37	C00966	2.47	0.0011
148.0772	[M+H]+	5-Methylthiopentanaldoxime	24785237	C17245	1.62	0.0391
160.0787	[M+Na]+	Tyramine	5408	C00483	1.84	0.0246
176.0718	[M+H]+	Indole-3-acetic acid	780	C00954	2.50	0.0340
177.0589	[M+H]+	N-Carbamoyl-L-aspartate	84022	C00438	2.56	0.0195
190.0550	[M+H]+	Kynurenic acid	3712	C01717	1.77	0.0219
190.1198	[M+H]+	8-Methylthiooctanaldoxime	24785473	C17251	2.23	0.0365
206.0541	[M+H]+	3-Indolylmethylthiohydroximate	24785009	C16516	1.71	0.0417
222.0351	[M+H]+	1-Chloromethyl-5-nitronaphthalene	196550		2.04	0.0267
229.0639	[M+Na]+	2-Methyl-1,8-naphthyridine-3-carboxylic acid hydrate	20172820		0.59	0.0393
244.0946	[M+H]+	Cytidine	5940	C00475	1.84	0.0041
251.0321	[M+Na]+	Mevalonate 5-phosphate	463	C01107	1.55	0.0119
252.0327	[M+Na]+	5-Phosphoribosylamine	388939	C03090	1.57	0.0022
271.0684	[M+Na]+	2-(5′-methylthio)pentylmalic acid	24784928	C17222	0.46	0.0148
277.2076	[M+Na]+	Palmitoleic acid	393216	C08362	1.59	0.0364
287.0513	[M+H]+	Kaempferol	4444395	C05903	2.12	0.0109
288.0614	[M+H]+	Cyanidin	114193	C05905	7.10	0.0079
289.0692	[M+H]+	Dihydrokaempferol	109514	C00974	2.08	0.0364
321.3134	[M+Na]+	Eicosanol	11898		1.92	0.0119
411.3584	[M+H]+	5-Dehydroavenasterol	10470275	C15783	1.78	0.0012
413.3764	[M+H]+	Stigmasterol	4444352	C05442	0.06	6.93x10^−6^
433.1115	[M+H]+	Vitexin	4444098	C01460	1.99	0.0493
611.1608	[M+H]+	Rutin	4444362	C05625	3.58	0.0175
62.0589	[M+H]+	Ethanolamine	13835336	C00189	0.49	0.0356
813.5053	[M+Na]+	trans-Nonaprenyl diphosphate	4444250	C04145	1.60	0.0091
90.0482	[M+H]+	L-Alanine	5735	C00041	1.68	0.0406

**Table 3:** Putatively identified metabolites with differential accumulation in aerial tissue after induction of the transcription factor. This includes the information of the *m*/*z* measured for each molecule, the theoretical form of ionization considered, the name in the ChemSpider database, the ID within this database, the corresponding ID in the KEGG database (if present in the database), the “fold change” (the ratio between the aerial induced (AI) and aerial control tissue (AC)), and the *p*-value.

*m/z*	Ionization Mode	Name in Chemspider	ID ChemSpider	ID KEGG	“Fold Change” (AI/AC)	*p*-Value
116.0723	[M+H]+	L-Proline	128566	C00148	1.77	3.73 × 10^−5^
175.1185	[M+H]+	L-Arginine	6082	C00062	1.87	8.90 × 10^−5^
413.3739	[M+H]+	Stigmasterol	4444352	C05442	0.11	0.0001
252.0327	[M+Na]+	5-Phosphoribosylamine	388939	C03090	1.55	0.0003
411.3584	[M+H]+	5-Dehydroavenasterol	10470275	C15783	1.72	0.0023
463.0952	[M+H]+	7-Methylthioheptyl glucosinolate	24785318	C17252	1.66	0.0071
321.3134	[M+Na]+	Eicosanol	11898		0.59	0.0072
277.2076	[M+Na]+	(Z)-Palmitoleic acid	393216	C08362	1.90	0.0147
431.3022	[M+H]+	Apocarotenoid	8725990		0.30	0.0199
465.3656	[M+H]+	Castasterone	117794	C15794	0.55	0.0234
419.3275	[M+Na]+	5-Dehydroepisterol	9069833	C15780	1.78	0.0383

**Table 4:** List of pathways in the KEGG database showing significant enrichment (*p* ≤ 0.05) among the differentially accumulated metabolites in root tissue.

Enriched Pathway (KEGG Database)	*p*-Value
Flavonoid biosynthesis	0.0012
Glucosinolate biosynthesis	0.0016
Alanine, aspartate and glutamate metabolism	0.0012
Tryptophan metabolism	0.0019
Terpenoid backbone biosynthesis	0.0022
Flavone and flavonol biosynthesis	0.0022
Phenylpropanoid biosynthesis	0.0030

**Table 5:** List of pathways in the KEGG database showing significant enrichment (*p* ≤ 0.05) among the differentially expressed genes in root tissue.

Enriched Pathway (KEGG Database)	*p*-Value
Phenylpropanoid biosynthesis	1.61 × 10^−5^
Zeatin biosynthesis	0.0001
leucine and isoleucine degradation	0.0001
Pentose and glucuronate interconversions	0.0001
Plant hormone signal transduction	0.0008
Glutathione metabolism	0.0013
ABC transporters	0.0016
Galactose metabolism	0.0017
Flavonoid biosynthesis	0.0043
Vitamin B6 metabolism	0.0044
Arginine and proline metabolism	0.0058
Protein processing in the endoplasmic reticulum	0.0059
DNA replication	0.0130
Cyanoamino acid metabolism	0.0205
Nitrogen metabolism	0.0212
Taurine and hypotaurine metabolism	0.0317
Monoterpenoid biosynthesis	0.0461

**Table 6:** (**A**) Differentially expressed genes and (**B**) differentially accumulated metabolites that belong to the phenylpropanoid (mostly flavonoid) biosynthetic pathway. The identifier used in the KEGG database for genes and metabolites, the abbreviated name of the genes, and the “fold change” between induced tissue and control tissue are shown.

ID	Name	“Fold Change” RI/RC
(**A**)		
AT1G67980	*CAFFEOYL-COA 3-O-METHYLTRANSFERASE (CCOAMT)*	3.23
AT3G51240	*TRANSPARENT TESTA 6* *(TT6)*	0.28
AT5G05270	*CHALCONE ISOMERASE LIKE* *(CHIL)*	0.26
AT5G08640	*FLAVONOL SYNTHASE 1* *(FLS1)*	0.11
AT5G13930	*TRANSPARENT TESTA 4(* *TT4)*	0.13
(**B**)		
C00974	Dihydrokaempferol	2.1
C01460	Vitexin	2
C05903	Kaempferol	2.1
C05905	Cyanidin	7.1
C05625	Rutin	3.6

**Table 7:** (**A**) Differentially expressed genes and (**B**) differentially accumulated metabolites identified within the glucosinolate biosynthetic pathway. The identifier used in the KEGG database for genes and metabolites, the abbreviated name of the genes, and the “fold change” between induced tissue and control tissue are shown.

ID	Name	“Fold Change” RI/RC
(**A**)		
AT1G10070	*BRANCHED-CHAIN AMINO ACID TRANSAMINASE 2 (BCAT-2)*	4.9363
AT2G43100	*ISOPROPYLMALATE ISOMERASE 2 (IPMI2)*	0.3209
AT4G13770	*REDUCED EPIDERMAL FLUORESCENCE 2(REF2*/*CYP83A1)*	0.2683
AT2G25450	*GLUCOSINOLATE HYDROXYLASE (GSL-OH)*	2.6089
AT3G09710	*IQ-DOMAIN 1 (IQD1)*	0.5063
AT3G44300	*NITRILASE 2 (NIT2)*	3.4643
AT5G22300	*NITRILASE 4 (NIT4)*	12.8733
(**B**)		
C16516	Indolylmethylthiohydroximate	1.7184
C17222	2-(5′-Methylthio)pentylmalate	0.4602
C17245	5-Methylthiopentanaldoxime	1.6259
C17251	8-Methylthiooctanaldoxime	0.0365

## Data Availability

Transcriptomic data is available at NCBI (BioProject ID PRJNA739193).

## Supplementary Materials

The following are available online at https://www.mdpi.com/article/10.3390/genes12070995/s1, Data S1: R code for metabolomic data analysis.
